# Anti-Proteinase 3 Anti-Neutrophil Cytoplasm Autoantibodies Recapitulate Systemic Vasculitis in Mice with a Humanized Immune System

**DOI:** 10.1371/journal.pone.0028626

**Published:** 2012-01-11

**Authors:** Mark A. Little, Bahjat Al-Ani, Shuyu Ren, Hamad Al-Nuaimi, Maurilo Leite, Charles E. Alpers, Caroline O. Savage, Jeremy S. Duffield

**Affiliations:** 1 Centre for Nephrology, Royal Free Hospital, University College London, London, United Kingdom; 2 Renal Institute of Birmingham, School of Infection, Immunology and Inflammation, University of Birmingham, Birmingham, United Kingdom; 3 Division of Nephrology, Department of Medicine, Center for Lung Biology and Institute for Stem Cell and Regenerative Medicine, University of Washington, Seattle, Washington, United States of America; 4 Division of Nephrology, Federal University of Rio de Janeiro, Rio de Janeiro, Brazil; 5 Department of Pathology, University of Washington, Seattle, Washington, United States of America; 6 GlaxoSmithKline UK Ltd, Uxbridge, United Kingdom; University of Pittsburgh, United States of America

## Abstract

Evidence is lacking for direct pathogenicity of human anti-proteinase-3 (PR3) antibodies in development of systemic vasculitis and granulomatosis with polyangiitis (GPA, Wegener's granulomatosis). Progress in study of these antibodies in rodents has been hampered by lack of PR3 expression on murine neutrophils, and by different Fc-receptor affinities for IgG across species. Therefore, we tested whether human anti-PR3 antibodies can induce acute vasculitis in mice with a human immune system. Chimeric mice were generated by injecting human haematopoietic stem cells into irradiated NOD-*scid*-*IL2Rγ^−/−^* mice. Matched chimera mice were treated with human IgG from patients with: anti-PR3 positive renal and lung vasculitis; patients with non-vasculitic renal disease; or healthy controls. Six-days later, 39% of anti-PR3 treated mice had haematuria, compared with none of controls. There was punctate bleeding on the surface of lungs of anti-PR3 treated animals, with histological evidence of vasculitis and haemorrhage. Anti-PR3 treated mice had mild pauci-immune proliferative glomerulonephritis, with infiltration of human and mouse leukocytes. In 3 mice (17%) more severe glomerular injury was present. There were no glomerular changes in controls. Human IgG from patients with anti-PR3 autoantibodies is therefore pathogenic. This model of anti-PR3 antibody-mediated vasculitis may be useful in dissecting mechanisms of microvascular injury.

## Introduction

Systemic vasculitis, comprising microscopic polyangiitis and granulomatosis with polyangiitis (GPA, Wegener's granulomatosis), is a debilitating and frequently life threatening disease affecting the microvasculature, in its most severe form manifesting with acute kidney and lung injury or failure. Its pathogenesis is only partly understood. Determining the mechanisms by which systemic vasculitis develops is paramount to developing new therapies that offer reduced toxicity. A major breakthrough was made in the 1980's when several groups discovered autoantibodies directed at cytoplasmic constituents of neutrophils (Anti-Neutrophil Cytoplasmic Antibodies [ANCA]) in patients affected by small vessel systemic vasculitides [1]–[5]. These antibodies, which have specificity for either neutrophil myeloperoxidase (MPO) or proteinase 3 (PR3), have been shown to activate primed neutrophils *in vitro* by binding to cell surface exposed antigens [6]–[7] and signalling via Fcγ receptors, suggesting a direct pathogenic role for the circulating antibodies. Further *in vitro* studies also lent support to a potential role for monocyte-ANCA interaction in vasculitis pathogenesis [8].

To study the pathogenicity of these antibodies further, investigators have attempted both passive transfer and active immunisation strategies to reproduce systemic vasculitis pathology in rodents [9]–[10]. A significant advance was made when Mpo deficient (*Mpo^−/−^*) mice were immunized with murine Mpo to generate anti-Mpo antibodies [11]–[14]. These studies confirmed a pathological role for mouse Mpo antibodies in mice, since passive transfer of anti-mouse Mpo antibodies to wild-type mice was sufficient to trigger mild vasculitis. However, the antibodies employed in this passive transfer model were raised in *Mpo^−/−^* mice that had never been exposed to the antigen before. Therefore, they are not autoantibodies and almost certainly have many different characteristics from antibodies that have arisen as a consequence of breakdown in tolerance and autoimmunity. A single report suggesting transfer of anti-MPO positive vasculitis from mother to neonate, presumably by placental transfer of IgG [15], provides further support for a pathogenic role for these antibodies, although it has never been proven in an experimental setting that human ANCA are sufficient to transfer disease.

Despite many attempts, studies have uniformly failed to develop a reliable model of anti-PR3 ANCA-associated vasculitis that proves anti-PR3 antibodies are necessary and sufficient to cause vasculitis [16]–[18]. The lack of pathogenicity of anti-PR3 antibodies in experimental systems may relate to three problems when crossing from humans to mice: 1) human antibodies do not recognize mouse neutrophil antigens; 2) mouse PR3 is not exposed at the surface of primed murine neutrophils, unlike on human neutrophils where it exhibits a bi-modal expression pattern [19]; 3) Human IgG does not bind to mouse Fcγ receptors. To address these issues we sought to test the function of anti-PR3 antibodies from patients with systemic vasculitis in mice that have circulating human neutrophils and monocytes.

## Results

### Human haematopoietic stem cells reconstitute the immune system of irradiated immunodeficient mice with human lymphocytes, monocytes and neutrophils

**Figure 1:**
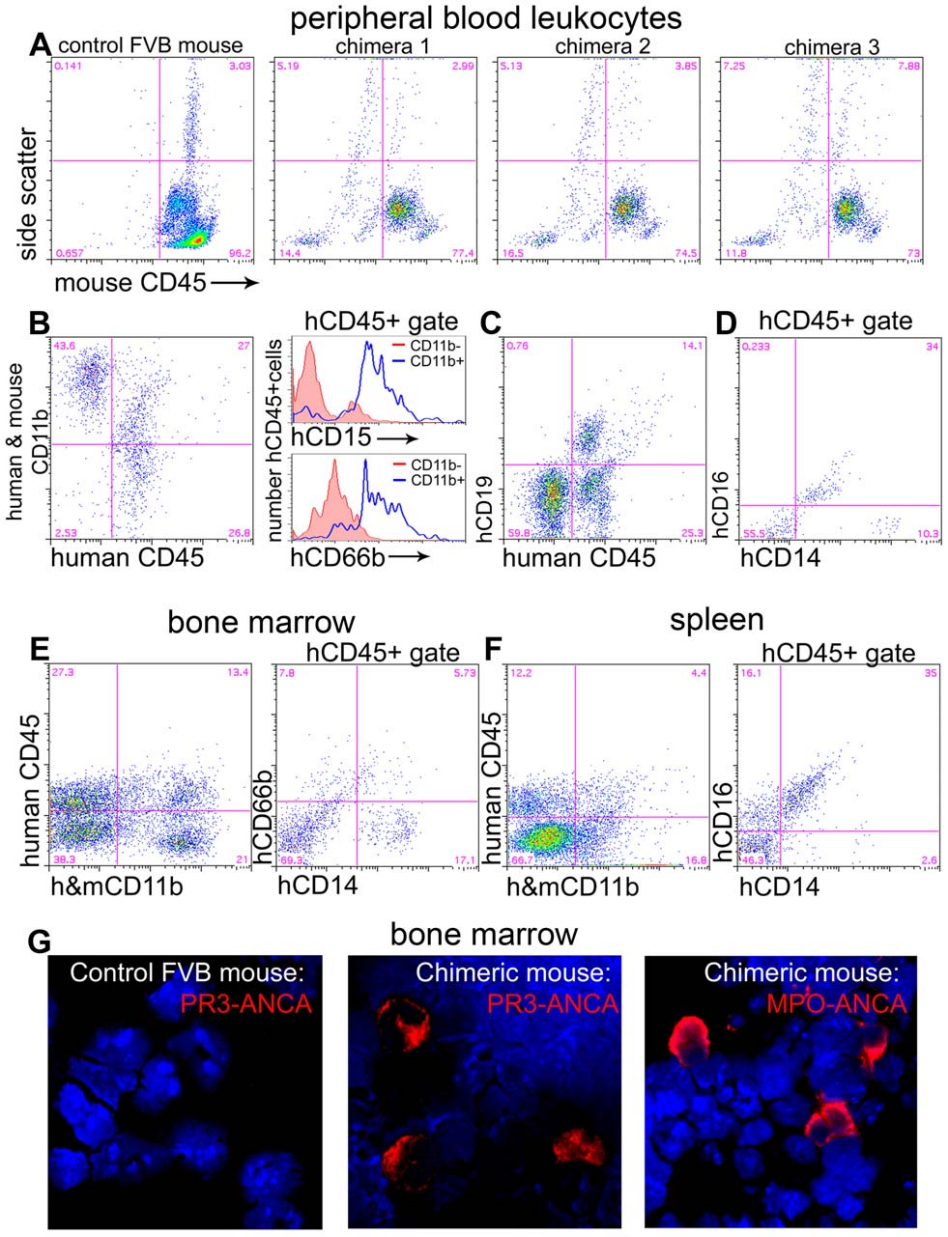
Characterization of chimerism in NOD-*scid*-*IL2Rγ^−/−^* mice. (A–D) Flow cytometric analysis of leukocytes from tail bleeds six weeks after administration of HSCs (n = 26 mice). (A) Plots showing mouse leukocytes labelled with anti-mouse CD45 antibodies. Compared with control wild-type mouse blood, chimeras have populations of mCD45 negative leukocytes that show SSC characteristics of granulocytes (High), monocytes (Int) and lymphocytes (low). (B) Chimera blood leukocytes express human CD45 and many of these express CD11b. hCD45+,CD11b+ leukocytes predominantly express hCD15 and hCD66b compared with hCD45+,CD11b− leukocytes shown in histograms. (C) A proportion of hCD45+ leukocytes express CD19. (D) Some hCD45 leukocytes are CD14^high^ and some are CD16+,CD14^low^. (E) In chimera bone marrow there are CD11b+ leukocytes which do not express mCD45 and among hCD45+ leukocytes a proportion express CD14 and a proportion express CD66b. (F) In chimera spleen there are CD11b+ leukocytes which express hCD45 and among hCD45+ leukocytes many express both CD14 and CD16. (G) Bone marrow spreads from wild type or chimera mice, labelled with anti-hMPO or anti-hPR3 IgG antibodies (red) purified from patients with vasculitis. Note that chimera bone marrow demonstrates anti-hMPO or anti-hPR3 antibody positive leukocytes with characteristic human neutrophil nuclear morphology. Wild type mouse bone marrow shows no cells positive for these antigens indicating that the anti-human antibodies do not cross react with mouse neutrophils.

We first sought to develop and characterise human-mouse chimeras by infusing mobilized CD34+ human bone marrow stem cells (HSCs) into sublethally irradiated NOD-*scid*-*IL2Rγ^−/−^* mice. None of the mice died following irradiation and stem cell injection. Six weeks after injection of HSCs the extent of chimerism of circulating leukocytes was assessed, initially by analysis of total blood leukocytes by flow cytometry. The extent of human chimerism was assessed by comparing the proportion of hCD45+ mCD45− leukocytes with total (human and mouse) CD45+ leukocytes (Table 1). In addition, we assessed the proportion of mCD45− leukocytes with high side scatter (SSC) (indicative of granulocytes) by flow cytometry compared with total high SSC cells to give a sense of the proportion of circulating granulocytes that were of human origin (Fig. 1A, Table 1) Once the degree of chimerism was established, mice were divided into experimental groups, matched for the degree of chimerism. The fraction of hCD45+/totalCD45+ cells was 24.2±2.7, 19.1±3.6, 17.9±2.4 in recipients of anti-PR3 IgG, disease control IgG and healthy control IgG respectively (*p* = NS). To determine more accurately the proportion of circulating human monocytes and neutrophils, blood leukocytes were labelled for expression of the myeloid marker CD11b (antibody recognizes both human and mouse CD11b) and the neutrophil restricted surface markers CD15 and CD66b, and analysed by flow cytometry. A significant population of hCD45+ CD11b+ hCD15+ leukocytes with high side scatter, or hCD45+ CD11b+ hCD66b+ leukocytes, with high side scatter was readily detected in all chimeras (Fig. 1B, Table 1), fulfilling all the criteria of circulating neutrophils. The proportion of human neutrophils as a fraction of total neutrophils was 17.8±1.6, 29.1±2.8, 49.1±11.6 in recipients of anti-PR3 IgG, disease control IgG and healthy control IgG respectively (*p* = NS). Next, we studied the proportion of circulating human monocytes using the markers CD14 and CD16 in addition to CD11b expression and high forward scatter (FSC) low SSC scatter characteristics. The human circulating CD11b+ leukocytes contained a population of CD16− CD14+ and CD16+ CD14^low^ cells with high FSC, low SSC, fulfilling all the criteria of human monocytes (Fig. 1D, Table 1). A large proportion of the human leukocytes expressed the B cell marker CD19 (Fig. 1C) and a minority were CD11b+ CD19− with scatter characteristics consistent with human NK cells (Table 1). CD3+ human T lymphocytes were rare but detectable.

**Table 1:** Characterization of the chimeric immune system.

	Blood	Bone marrow	Spleen
**% hCD45/total CD45**	**22.6±2.1**	**29.6±3.2**	**12.8±0.9**
**Monocytes**			
**% total hMono/hCD45**	**15.1±2.2**	**7.4±0.7**	
*% CD14high hMono/hCD45*	8.7±1.7		
*% CD14low hMono/hCD45*	6.3±2.7		
*% CD11b+CD14−/hCD45*			12.0±1.6
*% CD11b+CD14+/hCD45*			20.8±2.6
**Granulocytes**			
**% total hGranulo/hCD45**	**15.4±2.1**		
*% hCD66b+neutrophil/hCD45*	8.75±1.95		
*% CD16+ neutrophil/hCD45*	11.4±1.9		
*% hCD15+ neutrophil/hCD45*	10.8±2.1	3.8±0.4	
*% hGranulo/total Granulo*	33.0±3.9		
**Myeloid cells**			
*% CD11b+/hCD45*		47.1±5.2	
*% CD11b−/hCD45*		52.9±5.2	
**NK cells**			
**% hNK cells/hCD45**	**2.2±0.4**		
*%hCD56+ hNK cells/hCD45*			3.3±0.7
**T lymphocytes**			
**% hCD3/hCD45**	**0.34±0.10**		**1.9±0.3**
**B lymphocytes**			
**% hCD19+/hCD45**	**57.1±3.9**		**65.4±2.5**

Blood, bone marrow and splenic digests were analysed by flow cytometry to define the leukocyte populations (n = 26 mice). Treatment groups were matched by degree of peripheral blood chimerism prior to IgG injection. hMono = human monocytes. There was no significant difference in degree of chimerism or human granulocyte reconstitution between the experimental groups.

Next, we analysed spleen and bone marrow for chimerism by flow cytometry. In bone marrow, chimeras had human leukocytes of myeloid (CD11b+) and lymphoid (CD11b−) lineage (Fig. 1E, Table 1) and human bone marrow cells included CD14+ leukocytes and CD66b+ leukocytes in keeping with mature human neutrophils and monocytes residing in bone marrow (Fig. 1E, Table 1). In spleen, there was significant chimerism. Most human splenocytes were CD11b−, although there was a small population of CD16+ CD14^low^ myeloid cells (Fig. 1F, Table 1). Human CD3+ T cells and human CD56+ NK cells were also detected in spleen (Table 1).

In addition to cell surface markers by flow cytometry, human multi-lobed neutrophils were readily detected in the chimeric bone marrow spreads by incubating with anti-human PR3 hIgG or anti-human MPO hIgG derived from patients with systemic vasculitis and visualizing the binding with fluorescently conjugated anti-human IgG (Fig. 1G). Importantly, mouse myeloid cells are not detected by these antibodies (Fig. 1G).

### Preparation and characterization of human anti-PR3 immunoglobulin G

To determine the effect of the protein G-purified anti-PR3 antibodies on neutrophil function, we incubated IgG from healthy controls or patients with vasculitis with purified, tumour necrosis factor α (TNFα)-primed, human neutrophils. We determined the ability of each IgG preparation to stimulate neutrophil degranulation (as assessed by release of MPO from stimulated neutrophils) and release of superoxide (as assessed by reduction of ferricytochrome C in the presence of superoxide dismutase). On the basis of these experiments, we selected anti-PR3 IgG preparations that uniformly stimulated strong superoxide release in all neutrophil donors, and strong degranulation in two of the three preparations, reasoning that these would have the greatest likelihood of stimulating disease in mice (Table 2).

**Table 2:** Evidence of bioactivity of human anti-PR3 IgG on primed human neutrophils in vitro.

Stimulus	Superoxide release (AUC)	Degranulation (OD at 450 nm)	Creatinine (mg/dL)	Organs involved	BVAS
Anti-PR3-1 IgG	1236±122	0.35±0.06	1.3	K,L,S,N	26
Anti-PR3-2 IgG	1003±79	0.57±0.1	2.1	K,L	18
Anti-PR3-3 IgG	1180±120	0.13±0.03	9.1	K,L	20
Disease control IgG	291±40	0.12± 0.03	1.5		N/A
Healthy control IgG	161±16	0.13±0.02			N/A
fMLP	1378±153	1.06±0.10			N/A

IgG samples were incubated with primed human PMNs and superoxide release and degranulation were measured. Each assay was repeated at least 9 times using a minimum of 4 separate PMN donors. Organs involved: K = kidney, L = lung, S = skin, N = Nasal and sinus. Values represent mean±SEM.

### Passive transfer of human anti-PR3 ANCA into chimeric mice causes hematuria and macroscopic lung haemorrhage

In preliminary studies, lipopolysaccharide (LPS) or TNFα was administered to wild-type mice to determine their effect on neutrophil mobilization from bone marrow. Four hours after intra-peritoneal (IP) injection the proportion of CD45+ circulating leukocytes that expressed the neutrophil marker Ly6G was quantified by flow cytometry. LPS enhanced neutrophil recruitment to the peripheral circulation to a greater extent than TNFα (38.4±8.6% versus 18.9±6.1% of mCD45+ leukocytes respectively, *p*<0.05) (Table 3). In order to maximise the number of human neutrophils in the circulation at the time of antibody injection, and guided by prior investigations *in vitro* that indicated ANCA activity is markedly enhanced following neutrophil or endothelial exposure to TNFα or LPS, we determined to administer low-dose LPS to all groups of chimera mice prior to injection of human IgG in order to mobilize neutrophils into the circulation and increase the likelihood of triggering vasculitis [10]. To test whether human anti-PR3 IgG is capable of inducing systemic vasculitis, LPS-treated chimeric mice were injected next with anti-PR3 IgG or control IgG. Twenty four hours after administration of anti-PR3 IgG (n = 18 mice), disease control-IgG (n = 5 mice), or IgG from healthy controls (n = 3 mice), seven (39%) of the experimental mice that received anti-PR3 IgG had developed hematuria, whereas there was no hematuria in disease or healthy control animals (0%) (*p*<0.01). There was no difference in the capacity of anti-PR3 IgG purified from different patient donors to elicit disease (data not shown). In addition, experimental mice exhibited albuminuria (53.0±7.2 mg in anti-PR3 treated versus 36.7±17.9 mg in control animals, spot urine collection) at 24 h. All mice developed some albuminuria, which may be due to LPS administration. On d6 after injection of human IgG, mice were all observed to be alive with no signs of distress. The lungs of 13 of the experimental mice (72%), however, showed areas of focal pulmonary haemorrhage whereas all lungs of both control groups (n = 8) appeared normal (*p*<0.01). Skin, gut and other organs were macroscopically normal. Therefore, there was evidence of a systemic vasculitic process following injection of anti-PR3 IgG and LPS.

**Table 3:** Effect of LPS and TNFα on neutrophil recruitment into the mouse circulation.

	LY6G+ cell fraction in peripheral blood leukocytes		
*Compound*	Pre-treatment	4 hours	6 days
TNFα	13.6±3.7%	18.9±6.1%	11.1±3.2%
LPS	17.7±2.5%	38.4±8.6%*	10.1±1.5%

WT FVB mice were treated with TNFα (1 µg/kg i.v., n = 3) or LPS (1500 EU/g i.p., n = 3) and the degree of PMN mobilisation was quantified by staining peripheral blood for LY6G.

*p<0.05 compared to pre-treatment value. N = 3/group.

### Passive transfer of human anti-PR3 ANCA into chimeric mice causes acute glomerulonephritis

**Figure 2:**
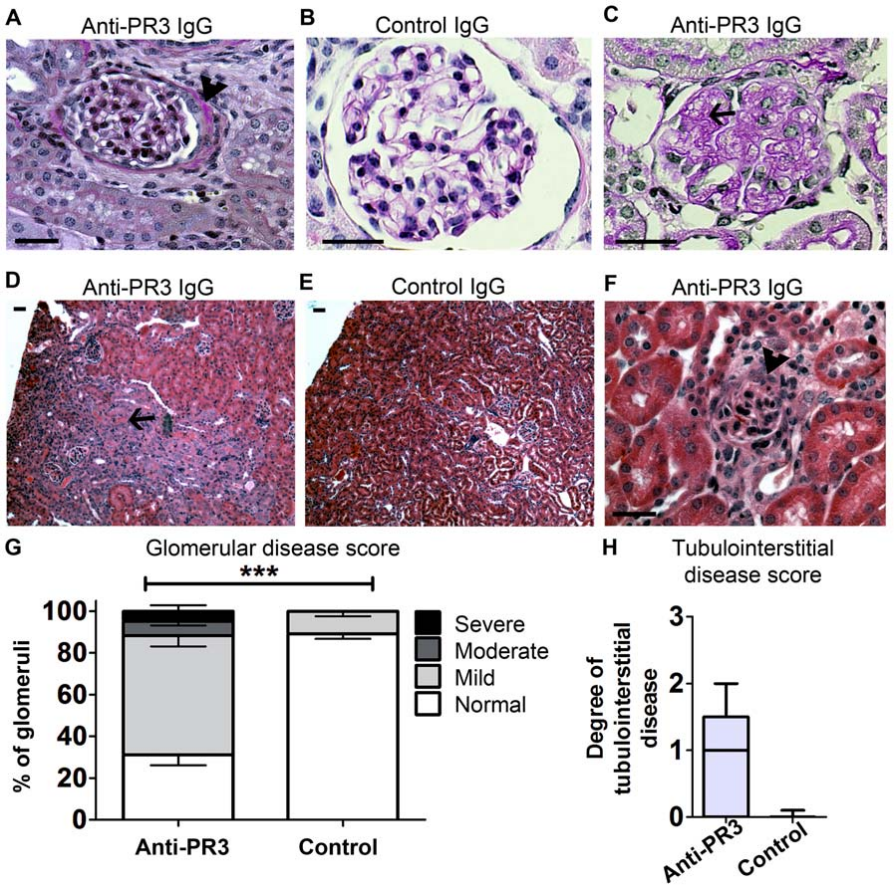
Anti-PR3 antibodies cause kidney disease. (A–C) PAS stained images of glomeruli from chimera mice 6 days after injection with anti-PR3 (n = 18, A, 400×; C, 600×) or control IgG (n = 8, B, 600×). Note extra-capillary proliferation and peri-glomerular inflammation (arrowhead) (A), and mesangiolysis (C, arrow) in anti-PR3 treated mice. (D–F) H & E stained sections of kidney from chimera mice treated with anti-PR3 (D, 40×) or disease control (E, 40×) IgG. There are regions of tubulointerstitial injury, with red cell cast formation (arrow). (F) Demonstrates intense peri-glomerular inflammation in an animal treated with anti-PR3 IgG (arrowhead, 400×). By comparison mice treated with disease control IgG showed minimal glomerular or tubulointerstitial changes. (G) Fractions of glomeruli affected in anti-PR3 (n = 18) and control IgG (n = 8) treated animals (Error bars depict SEM; ***p = 0.001) (H). Degree of tubulointerstitial disease in mice treated with anti-PR3 antibodies and control antibodies (*P<0.05, median ± IQ ± max/min values). (Bars = 50 µm).

**Figure 3:**
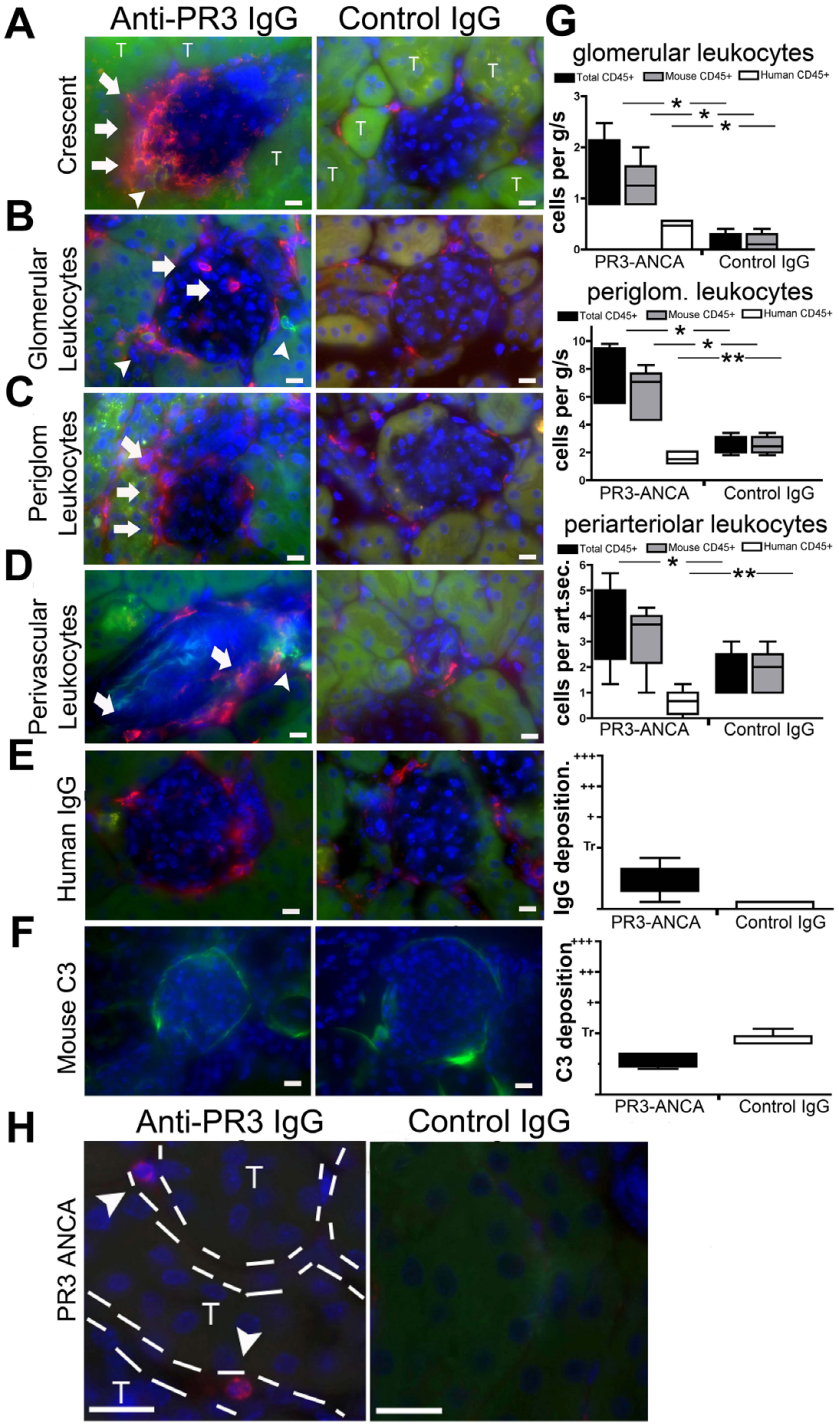
Anti-PR3 antibodies induce infiltration of kidneys with leukocytes of murine and human origin. Kidney sections were incubated with anti-mCD45 (red) and anti-hCD45 (green) antibodies and images were captured by fluorescence microscopy (T = tubule). Occasional (<5%) glomeruli of anti-PR3 treated mice displayed intense extracapillary leukocyte infiltration (A) in the shape of crescents (arrows). Most glomeruli in animals treated with anti-PR3 antibodies (n = 18) had evidence of intraglomerular (B,G) and peri-glomerular (C,G) leukocyte infiltration. These were comprised mostly of mCD45+ cells, although some hCD45 leukocytes were also present (arrowheads). In addition, there was a significant increase in peri-vascular leukocyte (mCD45+ and hCD45+) infiltration in anti-PR3 treated mice (D,G [per arteriolar section (art.sec.)]). Sections were also stained for deposition of IgG [red] (E,G) and C3 [green] (F,G). IgG was detectable within periglomerular cells, but there was minimal deposition within the glomeruli. Mouse C3 was weakly deposited in glomeruli but was no different between control group (n = 8) and anti-PR3 group (n = 18). Note mouse C3 can be detected normally binding avidly to tubular basement membranes. (Marker = 10 µm) (*P<0.05, **P<0.01. median ± IQ ± max/min values). (H) Kidney sections from anti-PR3 and control treated animals were incubated with anti-PR3 positive ANCA IgG. In the peritubular capillaries of chimera mice that received anti-PR3 hIgG occasional leukocytes detected by anti-hPR3 hIgG could be detected. No positively stained human neutrophils were seen in glomeruli.

Kidneys and lungs were examined by light microscopy after staining with hematoxylin & eosin (H&E) and periodic acid-Schiff (PAS). Fifteen (83%) of the mice treated with anti-PR3 IgG showed mild disease in kidneys with glomerular hypercellularity. In three (17%) of the anti-PR3 treated mice, glomeruli showed severe glomerular injury with proliferation and infiltration in Bowman's space of leukocytes, which were primarily mouse in origin (Fig. 2A–C, F–G & 3). Using standard histological stains the glomerular findings also comprised: glomerular tuft inflammation, areas of focal pyknosis, early glomerulosclerosis and periglomerular inflammation with matrix expansion. There was no significant difference in histological appearance between mice treated with anti-PR3 IgG preparations from each of the three patient donors (data not shown). Three (17%) of the mice treated with anti-PR3 IgG also showed evidence of moderate interstitial disease with tubule injury, with additional features of tubule haemorrhage, interstitial matrix expansion; and interstitial inflammation (Fig. 2D,E,H). The pathological changes were also evident in kidney cryosections labelled for leukocytes. Most glomeruli showed recruitment of mouse and human leukocytes to the glomerulus and periglomerular areas (Fig. 3A–D,G). In addition, there were leukocytes of both mouse and human origin in the interstitium of experimental but not control kidneys, and arterioles had notable perivascular leukocyte recruitment. Human (anti-hMPO and anti-hPR3+ve) neutrophils were detected in the renal interstitium of experimental but not control mice (Fig. 3H). Thus, there was pathological evidence of glomerulonephritis restricted to mice that had received anti-PR3 ANCA.

Human ANCA associated glomerulonephritis is described as ‘pauci-immune’, due to the lack of co-ordinated deposition of immune complexes along glomerular basement membrane or in mesangial or subendothelial spaces. Kidney sections were stained for the deposition of human IgG and mouse C3. Infiltrating leukocytes stained positive for human IgG in experimental and control kidneys, indicating that human IgG is endocytosed by interstitial cells, but glomeruli showed only weak deposition of IgG and C3 in all groups (Fig. 3E–G). These results are consistent with anti-PR3 IgG causing a ‘pauci-immune’ disease of the kidney of these mice.

### Passive transfer of human anti-PR3 ANCA into chimeric mice causes lung haemorrhage

**Figure 4:**
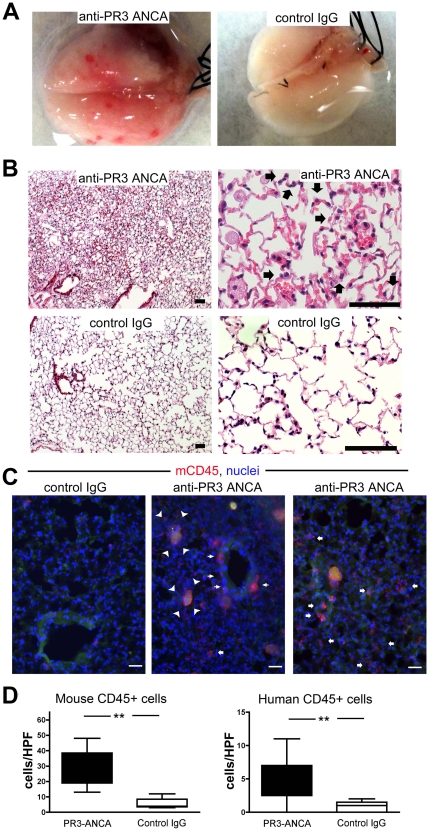
Anti-PR3 antibodies induce capillaritis with leukocytes of mouse and human origin. (A) Photomicrographs of explanted lungs from mice treated with disease control IgG (n = 8) or from patients with anti-PR3 ANCA (n = 18). Mice treated with anti-PR3 ANCA displayed petechiae over the surface of the lung. (B) H&E stained low and high power images of lungs from chimera mice injected with anti-PR3 antibodies 6 days previously. Note hemorrhage, inflammation, thickening of alveolar walls, enlarged, highly vacuolated alveolar macrophages and prominent recruitment of neutrophils (arrows) within the alveolar walls. These features are consistent with capillaritis. By comparison chimera mice treated with control IgG showed normal lung architecture. (C) Immunostaining of lung tissue to assess the degree of leukocyte infiltration. Note that in mice that received anti-PR3 ANCA IgG there was significant recruitment of leukocytes to the peribronchial areas (thin arrows) and also the alveolar areas (fat arrows). In addition many alveoli in mice treated with anti-PR3 antibodies had apoptotic debris that was partially ingested by alveolar macrophages (arrowheads) (bar = 50 µm). (D) Blinded assessment of human and mouse leukocyte recruitment to the lungs of treated animals (** *P*<0.01. median ± IQ ± max/min values).

Microscopic examination of the lungs from experimental mice showed patchy areas of haemorrhage with leukocyte recruitment, particularly of neutrophils to the alveolar walls, and thickening of alveolar walls. These microscopic features are consistent with capillaritis (Fig. 4A,B). Strikingly, there were peri-bronchiolar and peri-alveolar leukocytes in anti-PR3 treated animals, some with characteristic multi-lobed neutrophil nuclei. In addition, many alveolar spaces were filled with apoptotic and necrotic cellular debris, partially engulfed by enlarged alveolar macrophages (Fig. 4C). The number of leukocytes infiltrating into the lungs was significantly greater in anti-PR3 treated mice (Fig. 4D). As in the kidney, the majority of recruited leukocytes to the lung were of mouse origin. By comparison no leukocytes were recruited to the livers of these mice (data not shown).

## Discussion

These studies are the first to report a clear pathogenic role *in vivo* for IgG derived from patients with anti-PR3 antibody positive vasculitis. Anti-PR3 autoantibodies have the capacity to trigger all the hallmarks of vasculitis in mice with circulating human neutrophils, and prove therefore that anti-PR3 rich IgG is important in the pathogenesis of vasculitis. In addition, although rodent experiments have proven the ability of anti-MPO antibodies derived from MPO-immunised mice (without evidence of vasculitis) to induce systemic vasculitis, IgG from actual human patients with vasculitis has not previously been shown to transfer the pathological features of vasculitis to rodents. We have shown that it is possible to transfer disease from humans using antibody alone, thereby satisfying one of the key criteria for pathogenicity of these antibodies.

In order to study anti-PR3 antibodies *in vivo* we were required to generate mice with a humanized immune system since wild type mice have no pathogenic response to human anti-PR3 antibodies (data not shown). In addition, previous attempts to mirror the approach in murine anti-MPO vasculitis, whereby mice deficient in PR3 (and elastase) were used to raise antibodies to the target antigen, have been unsuccessful [16]. These findings therefore represent a new model to study ANCA associated vasculitis in rodents, and will be of use to the scientific community. The model may require further optimization since the extent of kidney disease was mild compared with lung disease. The extent of disease in these experiments may reflect the relatively low level of immune system chimerism and in particular the relatively low level of circulating human granulocytes. It is likely that higher levels of irradiation prior to stem cell administration, use of neonatal mice and more potent strategies for recruiting granulocytes from bone marrow will afford greater levels of myeloid chimerism in future studies. In previous reports using these NOD-*scid*-*IL2Rγ^−/−^* mice to generate human chimeras, the extent of peripheral blood myeloid cell chimerism in NOD-*scid*-*IL2Rγ^−/−^* mice was not well reported [20]. Here we show unequivocal myeloid chimerism in blood, bone marrow and spleen. The difference between these studies and earlier reports may be due to the use of Ficoll density gradients to purify leukocytes from mouse blood. Ficoll does not separate granulocytes from erythrocytes and so the extent of granulocytes in the circulation may have been underestimated previously. In these studies we exclusively used Cal-lyse which simply lyses all erythrocytes, leaving all leukocytes including granulocytes for analysis.

It was interesting to note that the majority of recruited leukocytes in the kidney and lung were of murine origin, although some infiltrating human leukocytes were observed. This suggests that the action of the anti-PR3 antibodies on the human neutrophils (and monocytes) in the chimeric mice led to the activation of “danger signals” in the recipient mice that led to recruitment of murine leukocytes in the first instance. The ability of human leukocytes to marginate, adhere to murine endothelium and transmigrate into inflamed tissue in these chimeric mice is questioned. Therefore, it is not surprising that relatively few human leukocytes were seen infiltrating the tissues. Indeed, the fact that we observed some, suggests that they do possess the capability to emigrate from vessels in response to local inflammatory stimuli and chemotactic agents in chimeric mice (presumably generated by murine cells). We observed human and murine leukocytes in close proximity in the glomerular lesions. It is thus likely that some cross talk between the leukocytes of the two species has occurred, an issue that will require further investigation.

In these proof of principle experiments we observed more severe lung injury than kidney injury, and no injury or leukocyte recruitment to other organs. These findings are similar to the findings in humans where anti-PR3 antibodies have a predilection for microvascular injury in lung and kidney [29]. Whether this strain of mice will be more susceptible to lung injury than kidney injury remains to be determined. Certainly, the studies of Primo *et al* suggest that the background strain of the immunodeficient mouse has an impact on the phenotype, as they observed a differential effect of transfer of splenocytes derived from murine PR3-immunised mice into mice of NOD and C57/Bl6 RAG−/− mice (the latter developed no disease) [21].

All the mice received LPS injections. This was performed to maximize neutrophil recruitment to the peripheral vasculature from bone marrow. In addition, LPS triggers endothelial activation which may be important in the initial trigger for priming neutrophils for degranulation by anti-PR3 antibodies [30]. However, the stimulus required to maximally trigger microvascular disease was not explored. It is not possible to determine from these studies therefore whether more severe disease can be triggered by passive transfer of antibodies alone. The chimera mice generated for these studies did not have CD3+ T lymphocytes of mouse origin and had few CD3+ T lymphocytes of human origin. Previous studies with this mouse showed presence of human CD3+ T lymphocytes, although these tended to develop later than the B cell population and their development in these mice was highly dependent on continuous administration of IL7-Fc fusion protein, which we did not administer [22]–[23] [20]. It is noteworthy that we did not identify any human T cells in the kidneys of the mice (not shown). Further studies, therefore, will be required to determine the role of human T lymphocytes in the pathogenesis of vasculitis in this mouse model, by generating chimeras and administering IL7-Fc, It is interesting to note, nevertheless, that vasculitis developed in the presence of very few T cells. It has been postulated that the reason previous anti-PR3 models have failed is because they lack effector T cells [24]; our results suggest that this is not a critical factor, although it is possible that the phenotype observed would be more severe in the presence of greater numbers of T cells.

In this report we have demonstrated a model of acute anti-PR3 antibody induced vascular injury that develops over a six-day period. In addition to this model of acute vasculitis, there is a great need for the development of models to study the granulomatous disease seen in GPA. Granulomata are dependent upon a robust cell mediated T cell response, the pathogenesis of which is almost certainly separate from the acute vasculitis observed in GPA, and which develop over a period of weeks or months. Hence, further refinement of the model to include greater levels of chimerism and administration of IL7-Fc protein to augment T cell development, will be required to study this cell mediated response.

To conclude, human anti-hPR3 antibodies from patients with ANCA associated pulmonary and renal vasculitis have the capacity to trigger all the features of vasculitis in mice with a humanized immune system. These findings are consistent with a model in which primed circulating neutrophils bind anti-PR3 antibodies to the neutrophil surface at the endothelial interface, with consequent triggering of neutrophil activation and degranulation, resulting in capillary injury and a subsequent immune response to the injured capillary. With further optimisation this model will allow in depth investigation of anti-PR3 associated vasculitis *in vivo*.

## Materials and Methods

All materials were purchased from Sigma Aldrich and all tissue culture supplies from Life Sciences unless otherwise stated.

### Preparation and selection of human IgG samples

Plasma was collected from the plasma exchange effluent of patients with active anti-PR3 positive pulmonary and renal vasculitis undergoing plasmapheresis as part of routine clinical care. Verbal consent was obtained, and clinical data were collected in a blinded, coded manner. The Birmingham University (UK) Ethics Review Board approved this study: UK research ethics number 5779 (Ethics Statement). All fulfilled Chapel Hill consensus classification criteria for diagnosis of systemic small vessel vasculitis [25]. Control IgG was purified from plasma exchange effluent derived from a patient with membranoproliferative glomerulonephritis (disease control) or pooled plasma from three healthy donors. Total IgG was separated from plasma using protein G affinity chromatography as detailed previously [26] and tested in degranulation and superoxide response assays as detailed previously [27]–[28]. In brief, superoxide production was determined at 37°C using a kinetic microplate assay. 96-well plates were incubated with 1×10^5^ Percoll purified neutrophils/well, 75 mM ferricytochrome C, 300 U/ml superoxide dismutase (SOD) and stimulus. IgG preparations were added at a concentration of 200 µg/ml and fMLP was used at 1 mM. Superoxide production was calculated using a molar extinction coefficient for ferricytochrome C of 21·1×10^3^ M^−1^ cm^−1^. Superoxide release was tracked for 120 minutes and the area under the curve was used as the comparator variable. Neutrophil degranulation was assessed by the release of myeloperoxidase as described previously [28]. In brief, neutrophils at 2.5×10^6^/ml were primed with TNFα and cytochalasin B and incubated with 200 µg/mL IgG for 15 min. Supernatants were removed and enzymatic activity was assessed by incubating them with MPO substrate for 30 minutes and assessing optical density at 405 nm. We chose the three IgG samples that induced the greatest superoxide or degranulation response for use in the *in vivo* experiments. These were derived during the first plasmapheresis session of patients with acute pulmonary and renal vasculitis (Birmingham Vasculitis Activity Score (BVAS) range: 18–26; mean serum creatinine level 4.2 mg/dL (range 1.3 to 9.1)).

### Generation and characterisation of chimeras

Mice with a partially human immune system were generated using female 8 week old NOD.Cg-Prkdc<scid> Il2rg<tm1Wjl>/SzJ (NOD-*scid*-*IL2Rγ^−/−^*) mice (Jackson laboratories), which lack native T, B and NK cells and the IL2 receptor [20]. Human stem cells were mobilized, purified and characterized from healthy donors as previously described [30]. Briefly, granulocyte colony stimulating factor (G-CSF) mobilized apheresis products obtained from normal healthy adult donors on d5 & 6 after stimulation were highly enriched for CD34+ cells from the apheresis product using ISOLEX 300i Magnetic Cell Positive Selection System (version 2.5, Baxter Healthcare, Deerfield, IL, USA). We used HSCs from a single donor for all of these studies, and mice were equally irradiated with a 300cG single dose over 20 mins and then injected by tail vein with 2×10^5^ mobilized human CD34+ hematopoietic stem cells. Mice were maintained in sterile conditions and monitored as previously described [29]. To quantify chimerism at six weeks, tail bleeds were performed to assess peripheral blood leukocytes by flow cytometry. Chimerism was defined as the proportion of hCD45+ mCD45− peripheral blood leukocytes compared with total (hCD45+ mCD45+) leukocytes. All mouse studies were performed according to protocols overseen and approved by the Animal Resources & Comparative Medicine at Harvard University (protocol numbers 04464 and 04231) (Ethics Statement).

### Flow Cytometry

**Purification of blood leukocytes**. In order to collect both granulocytes and PBMCs from mice the Cal-Lyse system (Invitrogen, CA, USA) was used. Tail bleeds (100 µl) or inferior vena cava (IVC) bleeds (500 µl) were treated with sodium citrate (0.38% final concentration). Aliquots were incubated with directly conjugated primary antibodies 15 min RT, then treated with one volume of Cal-lyse solution 10 min RT, followed by 10 volumes of ddH2O and vortexing. After centrifugation (350×*g* 5′) and washing in PBS, samples were resuspended in fluorescence activated cell sorting (FACS) buffer [31] and analysed directly using FACS-Calibur cytomteter. **Purification of Bone Marrow leukocytes**. Whole bone marrow (BM) was purified from long bones as previously described, subjected to red cell lysis, and resuspended in FACS buffer. **Purification of splenocytes**. One third of the spleen was dissociated by trituration as previously described [30] and single cells resuspended on ice in FACS buffer. 3×10^5^ BM cells or splenocytes were incubated on ice in 100 µl volume with primary antibodies. After washing and resuspension in FACS buffer they were fixed with 1% paraformaldehyde (PFA) solution and analysed using FACSCalibur cytometer. For controls, we analysed peripheral blood, BM and splenocytes from FVB/N WT mice. Human leukocytes were purified from a 2 ml blood sample from a healthy donor. Leukocytes were labelled with the following antibodies all at 1∶200 dilution: anti-hCD45-FITC, or –PE, hCD3-FITC, anti-hCD56-Al488 anti-hCD16-PE, anti-CD11b-APC, -PE or -FITC, anti-mCD45-FITC or -PE anti-mB220-APC (EBioscience), anti-hCD14-Alexa647, anti-hCD19-APC, anti-hCD15-FITC, anti-hCD66b-FITC (Biolegend), anti-Ly6C-FITC (BD Pharmingen). In blood, human granulocytes were defined as hCD45+ CD11b+ cells with high SSC. Neutrophils were defined as cells with these characteristics and the expression of hCD15, hCD66b or hCD16. Human monocytes were defined as hCD45+ CD11b+ cells with low SSC but high FSC. Subpopulations of monocytes were defined by the level of expression of CD14. Human NK cells were defined as hCD45+ hCD19−, hCD3− cells with low FSC and SSC characteristics or by expression of hCD56. Human B lymphocytes were defined as hCD45+ hCD19+ leukocytes with low FSC and SSC characteristics and human T lymphocytes were defined as hCD45+ hCD3+ cells with low FSC and SSC characteristics. In bone marrow, mature human neutrophils were defined as hCD45+ CD11b+ cells with high SSC and expression of hCD15. Mature human monocytes were defined as hCD45+ CD11b+ cells with low SSC but high FSC and co-expression of CD14.

### Passive transfer of human ANCA to mice

Unlike humans, many mature murine neutrophils and monocytes are held in bone marrow and only released in response to a stimulus. In preliminary studies the effect of TNFα or LPS (E. coli 026:B6) on mobilization of neutrophils from the BM of FVB/N mice into the circulation was studied. Mice were injected with vehicle, LPS (1500 EU/g/IP) or TNFα (1 µg/kg i.v.). Tail bleeds were performed pre-injection, at 4 hours and 6 days post injection (as described above) and mixed leukocytes were labelled with anti-CD45 and anti-Ly6G antibodies, then separated from RBCs with Cal-Lyse and analysed by flow cytometry. From these studies it was determined that LPS best stimulated neutrophil recruitment into the circulation. Chimera mice were separated into three matched groups based on the degree of chimerism. Baseline urine samples were collected. All mice received an IP injection of LPS (E. coli 026:B6) 1500 EU/g (100 µl). Thirty minutes later mice they received an IV injection of 4 mg of human IgG (200 µl) containing anti-PR3 antibodies from 1 of the 3 patients with lung and kidney vasculitis identified as having a potent effect *in vitro* on neutrophil degranulation and superoxide release (n = 18 mice), human IgG from patients with membranoproliferative glomerulonephritis (n = 5 mice) or human IgG from healthy volunteers (n = 3 mice). All 3 of the anti-PR3 patients contributed IgG to the experiment. Urine was collected on d1, d2, d4 and d6 and analysed for hematuria and proteinuria.

### Preparation of tissues for analysis

On d6 after injection, mice were euthanized and 500 µl blood obtained from the IVC. After flushing blood from the circulation, spleen and BM were harvested for cell analysis by FACS and in air-dried cell-spreads on slides [30]. Lungs, heart, trachea were removed together and inflated with paraformaldehyde-L-lysine-periodate (PLP) fixative using established methods to ensure preservation of lung architecture [32]. Kidneys, liver, small intestine, spleen and skin were collected and fixed with PLP prior to cryopreservation, or with 10% formalin for 12 h prior to dehydration with alcohols and paraffin embedding using standard methods.

### Immunostaining and pathological analysis of organs

Kidney and lung 3 µm paraffin sections were stained with PAS or H&E and analysed for glomerular and interstitial changes. Glomeruli were scored blindly as normal, mild glomerulonephritis (>4 cells per mesangial area or >4 cells per capillary loop), moderate glomerulonephritis (as per “mild” with evidence of necrosis or tuft disruption) or severe glomerulonephritis (as per “moderate” with extra-capillary or peri-glomerular proliferation). The interstitium was analysed for interstitial matrix expansion, inflammation and tubular epithelial cell injury and given a semi-quantitative score of 0–3 (0 = no injury, 1 = single focus of tubular injury or peri-tubular inflammation, 2 = >one focus of tubular injury with evidence of peri-tubular inflammation, 3 = extensive areas of tubular injury with loss of tubular integrity). Arterioles were examined for the presence of inflammatory cells. All scores used a 0–3 scale or % positivity as described previously [29], [33]. PLP fixed 5 µm cryosections were labelled with anti-mCD45 followed by affinity purified anti-rat IgG-Cy3 antibodies (1∶400, Jackson Immunoresearch), followed by anti-hCD45-FITC antibodies post-fixed with PFA1%, followed by mounting with Vectashield with DAPI, using methods previously described [34]. In some experiments, sections were labelled with anti-human IgG-Cy3 (Jackson Immunoresearch 1∶400), or anti-mouse C3-FITC (Cappell 1∶100). Glomeruli were scored blindly for deposition of IgG or C3 using a scale of 0, not detected; trace; +; ++; +++. In others, sections and bone marrow cell spreads were labelled with the anti-human PR3 ANCA IgG used in the transfer experiment (100 µg/mL) or anti-human MPO ANCA IgG (100 µg/mL) followed by anti-human IgG-Cy3 (1∶400).

### Statistical Analysis

Experimental conditions containing two groups were compared with the Mann Whitney U test or by ANOVA using Stat Plus (AnalysisSoft, USA) and those with >2 groups were compared using the Kruskal Wallis test.
